# Medical student wellbeing during COVID-19: a qualitative study of challenges, coping strategies, and sources of support

**DOI:** 10.1186/s40359-024-01618-8

**Published:** 2024-03-28

**Authors:** Helen M West, Luke Flain, Rowan M Davies, Benjamin Shelley, Oscar T Edginton

**Affiliations:** 1 Department of Psychology, University of Liverpool, Eleanor Rathbone Building, Bedford Street South, Liverpool, L69 7ZA UK; 2 Liverpool University Hospitals NHS Foundation Trust, Liverpool, UK; 3 School of Medicine, University of Liverpool, Liverpool, UK; 4 Salford Royal NHS Foundation Trust, Manchester, UK; 5 Calderdale and Huddersfield NHS Foundation Trust, West Yorkshire, UK; 6 Leeds Teaching Hospitals NHS Foundation Trust, Leeds, UK

**Keywords:** Mental health, Mental wellbeing, Medical student, Student doctor, COVID-19

## Abstract

**Background:**

Medical students face challenges to their mental wellbeing and have a high prevalence of mental health problems. During training, they are expected to develop strategies for dealing with stress. This study investigated factors medical students perceived as draining and replenishing during COVID-19, using the ‘coping reservoir’ model of wellbeing.

**Methods:**

In synchronous interactive pre-recorded webinars, 78 fourth-year medical students in the UK responded to reflective prompts. Participants wrote open-text comments on a Padlet site. Responses were analysed using reflexive thematic analysis.

**Results:**

Analysis identified five themes. COVID-19 exacerbated academic pressures, while reducing the strategies available to cope with stress. Relational connections with family and friends were affected by the pandemic, leading to isolation and reliance on housemates for informal support. Relationships with patients were adversely affected by masks and telephone consultations, however attending placement was protective for some students’ wellbeing. Experiences of formal support were generally positive, but some students experienced attitudinal and practical barriers.

**Conclusions:**

This study used a novel methodology to elicit medical students’ reflections on their mental wellbeing during COVID-19. Our findings reinforce and extend the ‘coping reservoir’ model, increasing our understanding of factors that contribute to resilience or burnout. Many stressors that medical students typically face were exacerbated during COVID-19, and their access to coping strategies and support were restricted. The changes to relationships with family, friends, patients, and staff resulted in reduced support and isolation. Recognising the importance of relational connections upon medical students’ mental wellbeing can inform future support.

**Supplementary Information:**

The online version contains supplementary material available at 10.1186/s40359-024-01618-8.

## Background

Medical students are known to experience high levels of stress, anxiety, depression and burnout due to the nature, intensity and length of their course [1]. Medical students are apprehensive about seeking support for their mental wellbeing due to perceived stigma and concerns about facing fitness to practise proceedings [2], increasing their vulnerability to poor mental health.

Research has identified that the stressors medical students experience include a demanding workload, maintaining work–life balance, relationships, personal life events, pressure to succeed, finances, administrative issues, career uncertainty, pressure around assessments, ethical concerns, and exposure to patient death [3, 4]. In March 2020, the COVID-19 pandemic introduced additional stressors into medical students’ lives. These included sudden alterations to clinical placements, the delivery of online teaching, uncertainty around exams and progression, ambiguity regarding adequate Personal Protective Equipment (PPE), fear of infection, and increased exposure to death and dying [5, 6]. Systematic reviews have reported elevated levels of anxiety, depression and stress among medical students during COVID-19 [7] and that the prevalence of depression and anxiety during COVID-19 was higher among medical students than in the general population or healthcare workers [8].

**Fig. 1:**
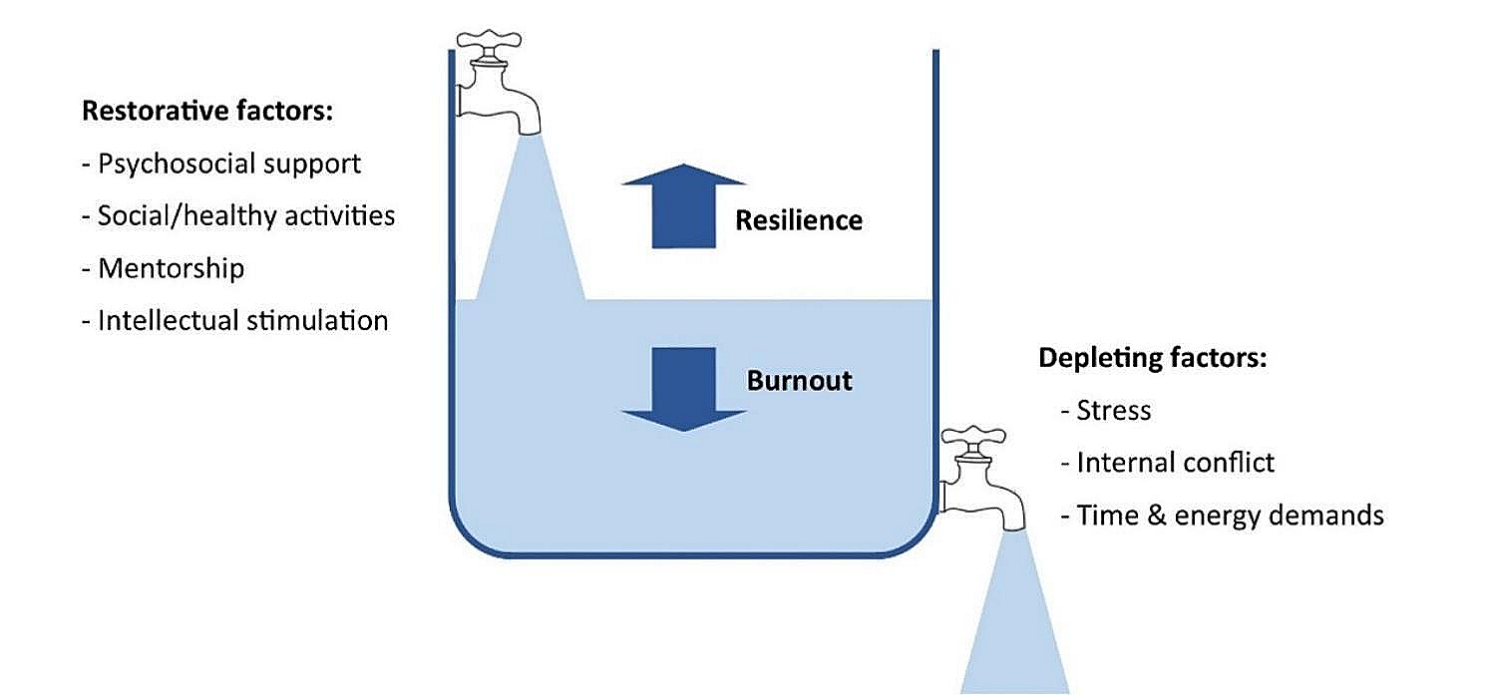
Coping reservoir model– adapted from Dunn et al. [14], with permission from the authors and Springer Nature

While training, medical students are expected to develop awareness of personal mental wellbeing and learn healthy coping strategies for dealing with stress [9]. Developing adaptive methods of self-care and stress reduction is beneficial both while studying medicine, and in a doctor’s future career. Protecting and promoting psychological wellbeing has the potential to improve medical students’ academic attainment, as well as their physical and mental wellbeing [10], and it is therefore important for medical educators to consider how mental wellbeing is fostered. Feeling emotionally supported while at medical school reduces the risk of psychological distress and burnout, and is related to whether students contemplate dropping out of medical training [11]. In their systematic narrative review of support systems for medical students during COVID-19, Ardekani et al. [12] propose a framework incorporating four levels: policies that promote a supportive culture and environment, active support for students at higher risk of mental health problems, screening for support needs, and provision for students wishing to access support. This emphasis on preventative strategies aligns with discussions of trauma-informed approaches to medical education, which aim to support student learning and prevent harm to mental wellbeing [13]. Dunn et al. [14] proposed a ‘coping reservoir’ model to conceptualise the factors that deplete and restore medical students’ mental wellbeing (Fig. 1). This reservoir is drained and filled repeatedly, as a student faces demands for their time, energy, and cognitive and emotional resources. This dynamic process leads to positive or negative outcomes such as resilience or burnout.

At present we have limited evidence to indicate why medical students’ mental wellbeing was so profoundly affected by COVID-19 and whether students developed coping strategies that enhanced their resilience, as suggested by Kelly et al. [15]. This study therefore sought to conceptualise the challenges medical students experienced during COVID-19, the coping strategies they developed in response to these stressors, and the supportive measures they valued. The ‘coping reservoir’ model [14] was chosen as the conceptual framework for this study because it includes both restorative and depleting influences. Understanding the factors that mediate medical students’ mental wellbeing will enable the development of interventions and support that are effective during crises such as the pandemic and more generally.

## Methods

### Methodology

This research study is based on a critical realist paradigm, recognising that our experience of reality is socially located [16]. Participant responses were understood to represent a shared understanding of that reality, acknowledging the social constructivist position that subjective meanings are formed through social norms and interactions with others, including while participating in this study. It also draws on hermeneutic phenomenology in aiming to interpret everyday experienced meanings for medical students during COVID-19 [17]. The use of an e-learning environment demonstrates an application of connectivism [18], a learning theory in which students participate in technological enabled networks. We recognise that meaning is co-constructed by the webinar content, prompts, ‘coping reservoir’ framework and through the process of analysis.

The multidisciplinary research team included a psychologist working in medical education, two medical students, and two Foundation level doctors. The team’s direct experience of the phenomenon studied was an important resource throughout the research process, and the researchers regularly reflected on how their subjective experiences and beliefs informed their interpretation of the data. Reflexive thematic analysis was chosen because it provides access to a socially contextualised reality, encompasses both deductive and inductive orientations so that analysis could be informed by the ‘coping reservoir’ while also generating unanticipated insights, and enables actionable outcomes to be produced [19].

### Ethical approval

Approval was granted by the University of Liverpool Institute of Population Health Research Ethics Committee (Reference: 8365).

### Participants

Fourth-year medical students at the University of Liverpool were invited to participate in the study during an online webinar in their Palliative Medicine placement. During six webinars between November 2020 and June 2021, 78 out of 113 eligible students participated, giving a response rate of 69%. This was a convenience sample of medical students who had a timetabled session on mental wellbeing. At the time, these medical students were attending clinical placements, however COVID-19 measures in the United Kingdom meant that academic teaching and support was conducted online, travel was limited, and contact with family and friends was restricted.

### Procedure

Students were informed about the study prior to the synchronous interactive pre-recorded webinar and had an opportunity to ask questions. Those who consented to participate accessed a Padlet (www.padlet.com) site during the webinar that provided teaching on mental wellbeing, self-care and resilience in the context of palliative medicine. Padlet is a collaborative online platform that hosts customisable virtual bulletin boards. During this recording, participants were asked to write anonymous open-text responses to reflective prompts developed from reviewing the literature (Appendix 1), and post these on Padlet. The Padlet board contained an Introduction to the webinar, sections for each prompt, links to references, and signposting to relevant support services. Data files were downloaded to Excel and stored securely, in line with the University of Liverpool Research Data Management Policy.

### Analysis

The research team used the six steps of reflexive thematic analysis to analyse the dataset. This process is described in Table 1, and the four criteria for trustworthiness in qualitative research proposed by Lincoln and Guba [20] are outlined in Table 2. We have used the purposeful approach to reporting thematic analysis recommended by Nowell et al. [21] and SRQR reporting standards [22] (Appendix 2).

**Table 1:** The process of reflexive thematic analysis in this study

1. Familiarisation with the dataset	Padlet responses were organised in an excel spreadsheet. The authors familiarised themselves with responses, noting any initial impressions and ideas.
2. Coding	Responses were systematically coded by BS & HMW, based on sematic similarities. Where participants had made multiple points within one posted comment these were separated so that each point could be coded individually (520 posts responding to 8 prompts were coded as 967 separate points). The researchers discussed these codes and differences of opinion with the wider team. It was noted that while a substantial portion of codes corresponded to the ‘coping reservoir’ conceptual framework (deductive coding), additional codes were also required (inductive coding).
3. Generating initial themes	Codes were grouped together to form provisional candidate themes by RMD, LF, OTE and HMW, adding notes on the impressions of data items. Candidate themes were discussed as a research team, particularly checking if they resonated with the experiences of researchers who were medical student and recent graduates.
4. Developing and reviewing themes	The researcher team reviewed and refined the provisional themes, revisiting the original dataset to check that the candidate themes presented an accurate summary of concepts that was grounded in the data. Themes were organised and reorganised until the team was satisfied that data were represented in a meaningful way.
5. Refining, defining and naming themes	OTE & HMW produced a synopsis of each theme, selecting data extracts that reflected the content. This was circulated to the other authors, who further refined the descriptions so that the scope and content of each theme was clear. Team consensus was sought for themes and names.
6. Writing up	The researcher team revised the written analysis, in line with the SRQR reporting standards [22] (Appendix 2), documenting the reasoning behind theoretical, methodological and analytical choices. Contextual descriptions of the themes were constructed, with a logical flow, embedding participants’ responses to illustrate the validity of the analysis.

**Table 2:** Strategies used to establish rigour and trustworthiness in this study [20]

Credibility	The credibility of analysis was enhanced by having several researchers code the dataset, develop, review and define themes, and produce the report. The multidisciplinary research team provided triangulation of perspectives and increases the credibility of the findings. The involvement of medical students and recent medical graduates was particularly important, as they confirmed whether interpretations of the dataset resonated with their lived experiences.
Transferability	We are aware that the cohort of students who participated were in a unique situation due to COVID-19. We have highlighted ways in which the themes are of continuing importance in medical education, and provided details of the study’s context for readers to evaluate whether the findings have transferability to other settings.
Dependability	We have reported the method of data collection and steps of reflexive thematic analysis in detail. Notes were kept of meetings and key decisions during analysis, to provide an audit trail of the process.
Confirmability	The researchers met regularly to discuss our interpretations of the data, with opportunities for reflection. The reasons for theoretical, methodological and analytical choices have been described using the SRQR reporting standards [22] (Appendix 2) to ensure that we transparently reported our findings.

## Results

Five themes were identified from the analysis:

- COVID-19 exacerbated academic pressures.
- COVID-19 affected students’ lifestyles and reduced their ability to cope with stress.
- COVID-19 changed relationships with family and friends, which affected mental wellbeing.
- COVID-19 changed interactions with patients, with positive and negative effects.
- Formal support was valued but seeking it was perceived as more difficult during COVID-19.

### COVID-19 exacerbated academic pressures

*‘Every day feels the same, it’s hard to find motivation to do anything.’*

Many participants reported feeling under chronic academic pressure due to studying medicine. Specific stressors reported were exams, revision, deadlines, workload, specific course requirements, timetables, online learning, placement, and communication from University. Some participants also reported negative effects on their mental wellbeing from feelings of comparison and competition, feeling unproductive, and overthinking.

*Massive amounts of work load that feels unachievable.*

COVID-19 exacerbated these academic stresses, with online learning and monotony identified as particularly draining. However, other students found online learning beneficial, due to reduced travelling.

I miss being able to see people face to face and zoom is becoming exhausting. My mental wellbeing hasn’t been great recently and I think the effects of the pandemic are slowly beginning to affect me.

*I also prefer zoom as it is less tiring than travelling to campus/placement.*

Clinical placements provided routine and social interaction. However, with few social interactions outside placement, this became monotonous. A reduction in other commitments helped some students to focus on their academic requirements.

*‘Most social activity only taking place on placement has made every day feel the same’.*

Some students placed high value on continuing to be productive and achieve academically despite the disruption of a pandemic, potentially to the detriment of their mental wellbeing. Time that felt unproductive was frustrating and draining.

‘Having a productive day i.e. going for a run and a good amount of work completed in the day’.

‘Unproductive days of revision or on placement’.

### COVID-19 affected students’ lifestyles and reduced their ability to cope with stress

*‘Everyone’s mental well-being decreased as things they used for mental health were no longer available’.*

Students often found it difficult to sustain motivation for academic work without the respite of their usual restorative activities challenging.

*‘Not being able to balance work and social life to the same extent makes you resent work and placement more’.*

The competing demands medical students encounter for their time and energy were repeatedly reported by participants.

*‘Sometimes having to go to placement + travel + study + look after myself is really tough to juggle!’*

However, removing some of the boundaries around academic contact and structure of extracurricular activities heightened the impact of stressors. Many participants focused on organising and managing their time to cope with this. Students were aware that setting time aside for relaxation, enjoyment, creativity, and entertainment would be beneficial for their wellbeing.

*‘Taking time off on the weekends to watch movies’.*

However, they found it difficult to prioritise these without feeling guilty or believing they needed to ‘earn’ them, and academic commitments were prioritised over mental wellbeing.

*‘Try to stop feeling guilty for doing something that isn’t medicine’.*

*‘Would like to say I’d do more to increase my mental wellbeing but finals are approaching and that will probably have to take priority for the next few months’.*

Medical students were generally aware that multiple factors such as physical activity, time with loved ones, spiritual care, nourishment and hobbies had a positive impact on their mental wellbeing. During COVID-19, many of the coping strategies that students had previously found helpful were unavailable.

*‘Initially it improved my mental well-being as I found time to care for myself, but with time I think everyone’s mental well-being decreased as things they used for mental health were no longer available e.g. gym, counselling, seeing friends’.*

Participants adapted to use coping strategies that remained available during the pandemic. These included walks and time spent outdoors, exercise, journaling, reflection, nutrition, and sleep.

‘Running’. ‘Yoga’. ‘Fresh air and walks’.

A few students also reported that they tried to avoid unhelpful coping strategies, such as social media and alcohol.

*‘Not reading the news, not using social media’.*

‘Avoiding alcohol as it leads to poor sleep and time wasted’.

Many participants commented on increased loneliness, anxiety, low mood, frustration, and somatic symptoms.

*‘Everyone is worn out and demotivated’.*

‘Feel that as I am feeling low I don’t want to bring others down’.

*‘Feel a lot more anxious than is normal and also easily annoyed and irritable.’*

However, not all students reported that COVID-19 had a negative effect on wellbeing. A small minority responded that their wellbeing had improved in some way.

*‘I think covid-19 has actually helped me become more self reliant in terms of well-being’.*

### COVID-19 changed relationships with family and friends, which affected mental wellbeing

*‘Family are a huge support for me and I miss seeing them and the lack of human contact.’*

Feeling emotionally supported by family and friends was important for medical students to maintain good mental wellbeing. However, COVID-19 predominantly had a negative impact on these relationships. Restrictions, such as being unable to socialise or travel during lockdowns, led to isolation and poor mental wellbeing.

‘Not being able to see friends or travel back home to see friends/family there’.

Participants frequently reported that spending too much time with people, feeling socially isolated, being unable to see people, or having negative social experiences had an adverse effect on their mental wellbeing. Relationships with housemates were a key source of support for some students. However, the increased intensity in housemate relationships caused tension in some cases, which had a particularly negative effect.

‘Much more difficult to have relationships with peers and began feeling very isolated’.

‘Talk about some of the experiences I’ve had on placement with my housemates’.

‘Added strain on my housemates to be the only ones to support me’.

Knowing that their peers were experiencing similar stressors helped to normalise common difficulties. The awareness that personal contacts were also struggling sometimes curtailed seeking informal support to avoid being a burden.

‘Actually discussing difficulties with friends has been most helpful, as it can sometimes feel like you’re the only one struggling, when actually most people are finding this year really difficult’.

‘Family and friends, but also don’t want to burden them as I know I can feel overwhelmed if people are always coming to me for negative conversations’.

### COVID-19 changed interactions with patients, with positive and negative effects

*‘With patients there has been limited contact and I miss speaking to patients.’*

Some students reported positive effects on relationships with patients, and feeling a sense of purpose in talking to patients when their families were not allowed to visit. Medical students felt a moral responsibility to protect patients and other vulnerable people from infection, which contributed to a reduction in socialising even when not constrained by lockdown.

‘Talking to patients who can’t get visitors has actually made me feel more useful’.

‘Anxiety over giving COVID-19 to patients or elderly relatives’.

Students occasionally reported that wearing PPE made interactions with patients more challenging. Students’ contact with patients changed on some placements due to COVID-19, for example replacing in-person appointments with telephone consultations, and they found this challenging and disappointing.

‘Masks are an impediment to meaningful connections with new people’.

‘GP block when I saw no patients due to it all being on the telephone’.

### Formal support was valued but seeking it was perceived as more difficult during COVID-19

*‘Feel a burden on academic and clinical staff/in the way/annoying so tend to just keep to myself.’*

Many participants emphasised the primary importance of support from family and friends, and their responses indicated that most had not sought formal support. While staff remained available and created opportunities for students to seek support, factors such as online learning and increased clinical workloads meant that some students found it harder to build supportive relationships with academic and placement staff and felt disconnected from them, which was detrimental for wellbeing and engagement.

‘Staff have been really helpful on placement but it was clear that in some cases, staff were overwhelmed with the workload created by COVID’.

*‘Even though academic staff are available having to arrange meetings over zoom rather than face to face to discuss any problem is off putting’.*

A few students described difficulty knowing what support was available, and identifying when they needed it.

‘It’s difficult to access support when you’re not sure what is available. Also you may feel your problems aren’t as serious as other people’s so hold off on seeking support’.

Formal support provided within the University included meetings with Academic Advisors, the School of Medicine wellbeing team, and University counselling service and mental health advisory team. It was also available from NHS services, such as GPs and psychological therapies. Those who had accessed formal support mostly described positive experiences with services. However, barriers to seeking formal support, such as perceived stigma, practicalities, waiting times for certain services, and concern that it may impact their future career were reported by some participants.

‘It is good that some services offer appointments that are after 5pm- this makes it more accessible to healthcare students’.

‘Had good experience with GPs about mental health personally’.

‘Admitting you need help or asking for help would make you look weak’.

‘Reassurance should be provided to medical students that accessing the wellbeing team is not detrimental to their degree. If anything it should be marketed as a professional and responsible thing to do’.

Some students preferred the convenience of remote access, others found phone or video impersonal and preferred in-person contact.

Students expressed that it was helpful when wellbeing support was integrated with academic systems, for example Academic Advisors or placement supervisors.

‘My CCT [primary-care led small group teaching] makes sure to ask how we are getting on and how our placements are going, so I think small groups of people with more contact with someone are more useful then large groups over zoom’.

‘Someone to speak to on palliative care placement, individual time with supervisor to check how we are doing (wellbeing, mental health) - would be a nice quick checkup’.

Participants typically felt able to share openly in an anonymous forum. Reading peers’ comments helped them to see that other students were having similar experiences and challenged unhealthy comparisons.

‘I definitely shared more than I would have done on a zoom call’.

‘I loved this session as it makes you feel like you’re not alone’.

‘Reassuring to know that there are others going through similar things as you’.

## Discussion

**Fig. 2:**
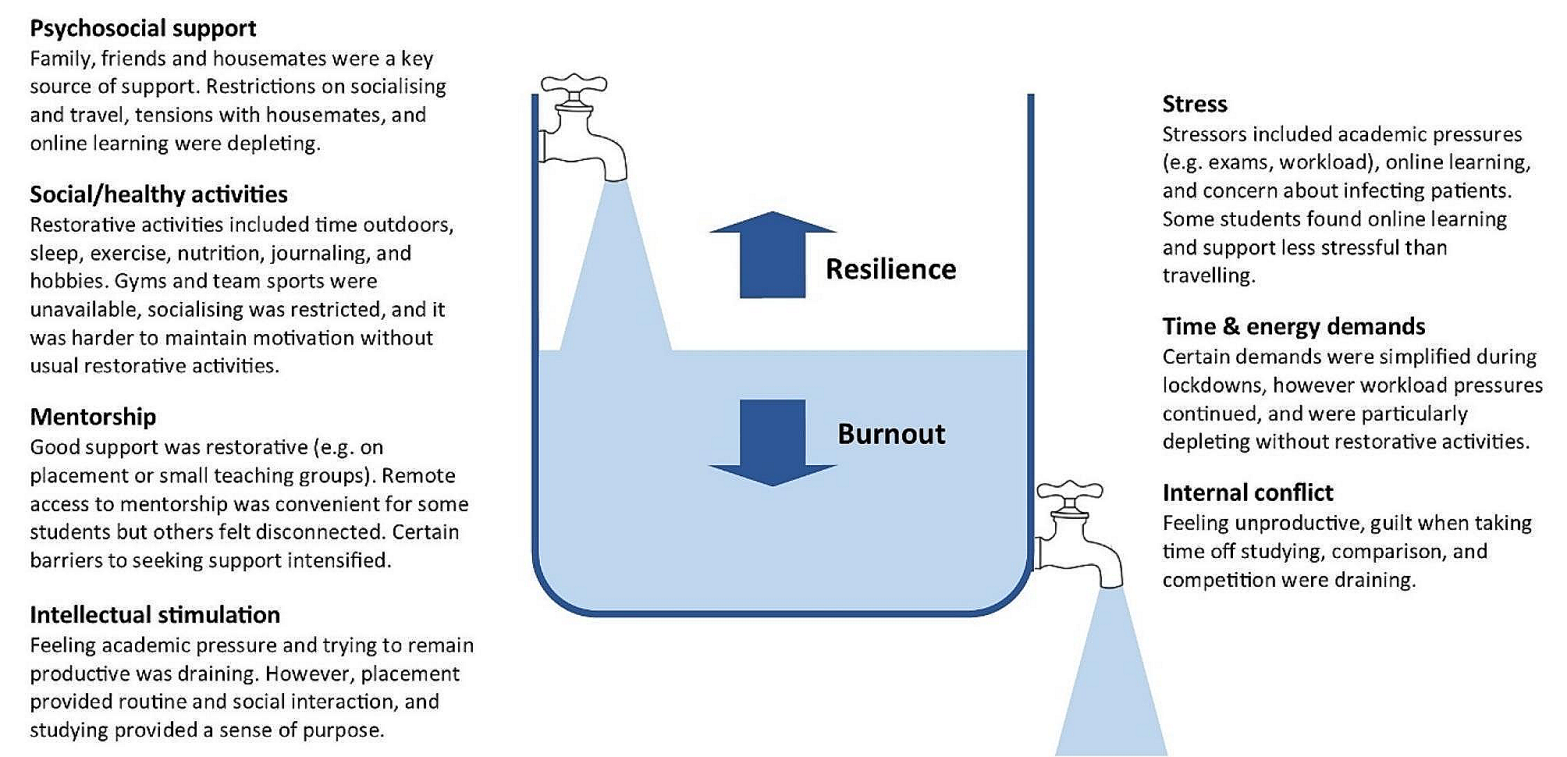
Coping reservoir model - the effects of COVID-19 on restorative and depleting factors for medical students, adapted from Dunn et al. [14], with permission from the authors and Springer Nature

Our findings demonstrate that the COVID-19 pandemic exacerbated the stressors medical students experience, and removed some rewarding elements of learning, while reducing access to pre-existing coping strategies. The results support many aspects of the ‘coping reservoir’ framework [14]. Findings corroborate the restorative effects of psychosocial support and social/healthy activities such as sleep and physical activity, and the depletion of wellbeing due to time and energy demands, stress, and disruptions relating to the pandemic such as online teaching and limited social interaction. Feeling a sense of purpose, from continuing studying or interactions with patients for example, was restorative for wellbeing. Mentorship and intellectual stimulation were present in the responses, but received less attention than psychosocial support and social/healthy activities. Internal conflict is primarily characterised by Dunn et al. [14] as ambivalence about pursuing a career in medicine, which was not expressed by participants during the study. However, participants identified that their wellbeing was reduced by feeling unproductive and lacking purpose, feeling guilty about taking time for self-care, competing priorities, and comparison with peers, all of which could be described as forms of internal conflict. Different restorative and draining factors appeared to not be equally weighted by the participants responding to the prompts: some appear to be valued more highly, or rely on other needs being met. Possible explanations are that students may be less likely to find intellectual stimulation and mentorship beneficial if they are experiencing reduced social support or having difficulty sleeping, and internal conflict about pursuing a career in medicine might be overshadowed by more immediate concerns, for example about the pandemic. This prioritisation resembles the relationship between physiological and psychological needs being met and academic success [23], based on Maslow’s hierarchy of needs [24]. A revised ‘coping reservoir’ model is shown in Fig. 2.

Relational connections with family, friends, patients, and staff were protective factors for mental wellbeing. Feeling emotionally supported by family and friends is considered especially important for medical students to maintain good mental wellbeing [11]. These relationships usually mitigate the challenges of medical education [25], however they were fundamentally affected by the pandemic. Restrictions affecting support from family and friends, and changes to contact with patients on placement, had a negative effect on many participants’ mental wellbeing. Wellbeing support changed during the pandemic, with in-person support temporarily replaced by online consultations due to Government guidelines. Barriers to seeking formal support, such as perceived stigma, practicalities, and concern that it may impact their future career were reported by participants, reflecting previous research [26]. Despite initiatives to increase and publicise formal support, some students perceived that this was less available and accessible during COVID-19, due to online learning and awareness of the increased workload of clinicians, as described by Rich et al. [27]. These findings provide further support for the job demand-resources theory [28, 29] where key relationships and support provide a protective buffer against the negative effects of challenging work.

In line with previous research, many participants reported feeling under chronic academic pressure while studying medicine [3]. Our findings indicate that medical students often continued to focus on achievement, productivity and competitiveness, despite the additional pressures of the pandemic. Remaining productive in their studies might have protected some students’ mental wellbeing by providing structure and purpose, however students’ responses primarily reflected the adverse effect this mindset had upon their wellbeing. Some students felt guilty taking time away from studying to relax, which contributes to burnout [30]^,^and explicitly prioritised academic achievement over their mental wellbeing.

Students were aware of the factors that have a positive impact on their mental wellbeing, such as physical activity, time with loved ones, spiritual care, nourishment and hobbies [31]. However, COVID-19 restrictions affected many replenishing factors, such as socialising, team sports, and gyms, and intensified draining factors, such as academic stressors. Students found ways to adapt to the removal of most coping strategies, for example doing home workouts instead of going to the gym, showing how they developed coping strategies that enhanced their resilience [15]. However, they found it more difficult to mitigate the effect of restrictions on relational connections with peers, patients and staff, and this appears to have had a particularly negative impact on mental wellbeing. While clinical placements provided helpful routine, social interaction and a sense of purpose, some students reported that having few social interactions outside placement became monotonous.

Our findings show that medical students often felt disconnected from peers and academic staff, and reported loneliness, isolation and decreased wellbeing during COVID-19. This corresponds with evidence that many medical students felt isolated [32], and students in general were at higher risk of loneliness than the general population during COVID-19 lockdowns [33]. Just as ‘belongingness’ mediates subjective wellbeing among University students [34], feeling connected and supported acts as a protective buffer for medical students’ psychological wellbeing [25].

### Translation into practice

Based on the themes identified in this study, specific interventions can be recommended to support medical students’ mental wellbeing, summarised in Table 3. This study provides evidence to support the development of interventions that increase relational connections between medical students, as a method of promoting mental wellbeing and preventing burnout. Our findings highlight the importance of interpersonal relationships and informal support mechanisms, and indicate that medical student wellbeing could be improved by strengthening these. Possible ways to do this include encouraging collaboration over competition, providing sufficient time off to visit family, having a peer mentor network, events that encourage students to meet each other, and wellbeing sessions that combine socialising with learning relaxation and mindfulness techniques. Students could be supported in their interactions with patients and peers by embedding reflective practice such as placement debrief sessions, Schwartz rounds [35] or Balint groups [36], and simulated communication workshops for difficult situations.

**Table 3:** Suggested interventions to translate themes into practice

Theme	Suggested implications for practice
COVID-19 exacerbated academic pressures	• Manageable workloads • Supportive learning environments • Encouragement to take breaks without guilt • Cultivating students’ sense of purpose
COVID-19 affected students’ lifestyles and reduced their ability to cope with stress	• Promote healthy lifestyle habits, reflection, time management, mindset • Normalise sharing difficulties • Integrated content on self-care and managing stress
COVID-19 changed relationships with family and friends, which affected mental wellbeing	• Encourage collaboration over competition • Peer mentor and buddy schemes • Sufficient time to visit family • Opportunities to meet peers with similar interests • Including a social element with other wellbeing content e.g. relaxation and mindfulness
COVID-19 changed interactions with patients, with positive and negative effects	• Placement debrief sessions • Schwartz rounds • Balint groups • Simulated communication workshops
Formal support was valued but seeking it was perceived as more difficult during COVID-19	• Continue to address stigma around seeking support • Wellbeing checks integrated into routine academic systems • Responsive, individual and accessible support • Options for in-person and online access

Experiencing guilt [30] and competition [4] while studying medicine are consistently recognised as contributing to distress and burnout, so interventions targeting these could improve mental wellbeing. Based on the responses from students, curriculum-based measures to protect mental wellbeing include manageable workloads, supportive learning environments, cultivating students’ sense of purpose, and encouraging taking breaks from studying without guilt. Normalising sharing of difficulties and regularly including content within the curriculum on self-care and stress reduction would improve mental wellbeing.

In aiming to reduce psychological distress among medical students, it is important that promotion of individual self-care is accompanied by reducing institutional stressors [11, 29]. While the exploration of individual factors is important, such as promoting healthy lifestyle habits, reflection, time management, and mindset changes, this should not detract from addressing factors within the culture, learning and work environment that diminish mental wellbeing [37]. Heath et al. [38] propose a pro-active, multi-faceted approach, incorporating preventative strategies, organisational justice, individual strategies and organisational strategies to support resilience in healthcare workers. Similarly, trauma-informed medical education practices [13] involve individual and institutional strategies to promote student wellbeing.

Students favoured formal support that was responsive, individualised, and accessible. For example, integrating conversations about wellbeing into routine academic systems, and accommodating in-person and remote access to support. There has been increased awareness of the wellbeing needs of medical students in recent years, especially since the start of the pandemic, which has led to improvements in many of these areas, as reported in reviews by Ardekani et al. [12] and Klein and McCarthy [39]. Continuing to address stigma around mental health difficulties and embedding discussions around wellbeing in the curriculum are crucial for medical students to be able to seek appropriate support.

### Strengths & limitations

By using qualitative open-text responses, rather than enforcing preconceived categories, this study captured students’ lived experience and priorities [4, 31]. This increased the salience and depth of responses and generated categories of responses beyond the existing evidence, which is particularly important given the unprecedented experiences of COVID-19. Several strategies were used to establish rigour and trustworthiness, based on the four criteria proposed by Lincoln and Guba [20] (Table 2). These included the active involvement of medical students and recent medical graduates in data analysis and the development of themes, increasing the credibility of the research findings.

Potential limitations of the study are that participants may have been primed to think about certain aspects of wellbeing due to data being collected during a webinar delivered by medical educators including the lead author at the start of their palliative medicine placement, and the choice of prompts. Data was collected during the COVID-19 pandemic, and therefore represents fourth year medical students’ views in specific and unusual circumstances. Information on this context is provided to enable the reader to evaluate whether the findings have transferability to their setting. Responses were visible to others in the group, so participants may have influenced each other to give socially acceptable responses. This process of forming subjective meanings through social interactions is recognised as part of the construction of a shared understanding of reality, and we therefore view it as an inherent feature of this methodology rather than a hindrance. Feedback on the webinar indicated that students benefitted from this process of collective meaning-making. Similarly, researcher subjectivity is viewed as a contextual resource for knowledge generation in reflexive thematic analysis, rather than a limitation to be managed [19]. The study design meant that different demographic groups could not be compared.

Padlet provided a novel and acceptable method of data collection, offering researchers and educators the potential benefits of an anonymous forum in which students can see their peers’ responses. The use of an interactive webinar demonstrated a potential application of connectivist pedagogical principles [18]. Researchers are increasingly using content from online forums for qualitative research [40], and Padlet has been extensively used as an educational tool. However, to the authors’ knowledge, Padlet has not previously been used as a data collection platform for qualitative research. Allowing anonymity carried the risk of students posting comments that were inappropriate or unprofessional. However, with appropriate guidance it appeared to engender honesty and reflection, provided a safe and collaborative learning environment, and student feedback was overwhelmingly positive. It would be useful to evaluate the effects of this reflective webinar on medical students’ mental wellbeing, given that it acted as an intervention in addition to a teaching session and research study.

Students were prompted to plan what they would do following the webinar to improve their mental wellbeing. A longitudinal study to determine how students enacted these plans would allow a more detailed investigation of students’ self-care behaviour.

## Conclusions

While we hope that the stressors of COVID-19 will not be repeated, this study provides valuable insight into medical students’ mental wellbeing, which can inform support beyond this exceptional time. The lasting impact of the pandemic upon medical education and mental wellbeing remains to be seen. Nevertheless, our findings reinforce and extend the coping reservoir model proposed by Dunn et al. [14], adding to our understanding of the factors that contribute to resilience or burnout. In particular, it provides evidence for the development of interventions that increase experiences of relational connectedness and belonging, which are likely to act as a buffer against emotional distress among medical students.

### Electronic supplementary material

Below is the link to the electronic supplementary material.

Supplementary Material 1

Supplementary Material 2

## Data Availability

The datasets generated and analysed during the study are available from the corresponding author on reasonable request.
