# Publisher Correction: Medical student wellbeing during COVID-19: a qualitative study of challenges, coping strategies, and sources of support

**DOI:** 10.1186/s40359-024-01813-7

**Published:** 2024-06-26

**Authors:** Helen M West, Luke Flain, Rowan M Davies, Benjamin Shelley, Oscar T Edginton

**Affiliations:** 1 Department of Psychology, University of Liverpool, Eleanor Rathbone Building, Bedford Street South, Liverpool, L69 7ZA UK; 2 Liverpool University Hospitals NHS Foundation Trust, Liverpool, UK; 3 School of Medicine, University of Liverpool, Liverpool, UK; 4 Salford Royal NHS Foundation Trust, Manchester, UK; 5 Calderdale and Huddersfield NHS Foundation Trust, West Yorkshire, UK; 6 Leeds Teaching Hospitals NHS Foundation Trust, Leeds, UK

**Publisher Correction: BMC Psychology (2024) 12:179**

10.1186/s40359-024-01618-8

Following publication of the original article, it came to the journal’s attention that as a result of processing errors during production of the article, there was incorrect or inconsistent formatting present throughout the published article. In particular, italics, quotations marks, and line breaks were not used consistently for the participant quotations. The formatting has since been corrected in the article. The publisher thanks you for reading and apologizes for any inconvenience caused.

