# Supplementary material for: Medical student wellbeing during COVID-19: a qualitative study of challenges, coping strategies, and sources of support
Source: BMC Psychol. 2024 Mar 28;12:179. doi: 10.1186/s40359-024-01618-8 (PMC10979564; doi:10.1186/s40359-024-01618-8)
Supplement: Supplementary file 1 — Supplementary Material 1 [file 40359_2024_1618_MOESM1_ESM.docx]

**Appendix 1: Reflective prompts**

- One-word check-in: How do you feel at the moment?
- Experiencing stress: What are your ‘warning signs’ that stress is becoming unmanageable?
- Mental health reservoir: What refreshes and restores you?
- Mental health reservoir: What drains and depletes you?
- What steps will you take during this placement to increase your resilience and mental wellbeing?
- Relational connections:
  - How has COVID-19 affected your relationships with peers, patients, and academic staff?
  - Do you feel that these changes have affected your mental wellbeing, and if so, in what ways?
- Sources of support:
  - What support (if any) do you think would be helpful to you?
  - What makes it easier or more difficult for you to seek or access support?
- Lecture feedback: What worked well? What could be improved?
